# Systemic Inflammation Characterizes Lack of Metabolic Health in Nonobese HIV-Infected Men

**DOI:** 10.1155/2018/5327361

**Published:** 2018-09-25

**Authors:** Ana N. Monczor, Xiuhong Li, Frank J. Palella, Kristine M. Erlandson, Dorothy Wiley, Lawrence A. Kingsley, Wendy S. Post, Lisa P. Jacobson, Todd T. Brown, Jordan E. Lake

**Affiliations:** ^1^McGovern Medical School at The University of Texas Health Science Center at Houston, Houston, TX, USA; ^2^Johns Hopkins University, Baltimore, MD, USA; ^3^Northwestern University, Chicago, IL, USA; ^4^University of Colorado Denver, Denver, CO, USA; ^5^University of California Los Angeles, Los Angeles, CA, USA; ^6^University of Pittsburgh, Pittsburgh, PA, USA

## Abstract

**Background:**

Increasing body mass index (BMI) is generally associated with loss of metabolic health, although some obese individuals remain metabolically healthy. Among nonobese men, HIV infection has been associated with a lower prevalence of metabolic health.

**Methods:**

We conducted a cross-sectional analysis of 470 HIV-infected and 368 HIV-uninfected men enrolled in the Multicenter AIDS Cohort Study Cardiovascular substudy. Circulating biomarker levels were compared by BMI category and by HIV serostatus. Poisson regression with robust variance determined associations between metabolic health and circulating inflammatory biomarker levels after adjusting for factors previously associated with metabolic health.

**Results:**

HIV-infected men were younger and less likely to be obese. Among HIV-infected, normal weight metabolically healthy men (compared to unhealthy) had significantly lower circulating levels of interleukin- (IL-) 6, soluble tumor necrosis factor receptors (sTNFR) I and II, and homeostatic model assessment of insulin resistance (HOMA-IR), higher adiponectin, less visceral fat, and more subcutaneous fat. Among HIV-uninfected normal weight men and obese men (regardless of HIV serostatus), metabolic health was associated only with higher levels of adiponectin, less visceral fat, and lower HOMA-IR values. In multivariate analyses restricted to HIV-infected men, lower hs-CRP, sTNFRI, sTNFRII, and HOMA-IR and higher adiponectin levels were associated with metabolic health. Additional adjustment for visceral adiposity did not alter results.

**Conclusions:**

Among HIV-infected normal weight men, metabolic health was associated with less systemic inflammation, a relationship that, among normal weight men, was unique to HIV+ men and did not exist among obese men of either HIV serostatus.

## 1. Introduction

Adipose tissue (AT) dysfunction is tightly linked to both traditional cardiovascular (CVD) risk factors and HIV- and antiretroviral treatment- (ART-) specific contributors [1–5] and may significantly impact morbidity and mortality in persons living with HIV [6, 7]. AT disturbances, including generalized obesity and visceral fat accumulation, are common in the modern ART era and differ from those of classic HIV-associated lipodystrophy. Some cohorts report the frequency of BMI ≥25 kg/m^2^ among people living with HIV to be 42–65% [8, 9]. Since HIV infection and obesity are both chronic inflammatory states, their coexistence may hasten metabolic dysfunction.

Regardless of HIV serostatus, with weight gain, AT depots can expand by hyperplasia, the generation of new uniformly sized adipocytes, or by hypertrophy, where existing adipocytes become larger, less well differentiated, and lipid engorged [10, 11]. Hyperplasia is considered a healthier form of expansion that does not induce proinflammatory tissue-level changes. Conversely, hypertrophy results in decreased adiponectin concentrations, increased levels of leptin, interleukin- (IL-) 6, tumor necrosis factor- (TNF-) *α*, and IL-1*β* concentrations, an influx of type 1 proinflammatory macrophages into AT, tissue hypoxia, and adipocyte necrosis [12]. Together, the immuno-metabolic disturbances accompanying AT hypertrophy lead to insulin resistance. Furthermore, HIV infection and antiretroviral drug use may promote AT generation and enhanced AT inflammation [13, 14]. The sequelae of these processes include suppressed adiponectin concentrations, which is related to the severity of subclinical atherosclerosis among HIV-infected men, independent of traditional CVD risk factors [15].

In the general population, a state of metabolically healthy obesity (MHO) has been described. MHO is obesity without overt concomitant cardiometabolic disease and has been variably defined to include persons with BMI ≥ 25 or 30 kg/m^2^ and, most commonly, two or fewer metabolic syndrome criteria [16]. However, the definition of metabolic health remains controversial [6]. We previously documented, among men enrolled in the Multicenter AIDS Cohort Study (MACS), that the prevalence of MHO did not vary by HIV serostatus [17]. Additionally, among normal weight men, men living with HIV were significantly less likely than HIV-uninfected men in the same BMI category to be metabolically healthy.

As a next step, we evaluated circulating concentrations of biomarkers of inflammation and AT function, metabolic phenotypes, and BMI category to determine associations between metabolic health status and levels of systemic inflammation by HIV serostatus. We hypothesized that metabolically unhealthy men would have higher circulating levels of systemic inflammation, immune activation, and insulin resistance and lower adiponectin concentrations than metabolically healthy men at all BMI categories. We also postulated that obese participants would have higher circulating levels of markers of systemic inflammation, immune activation, and insulin resistance and lower adiponectin concentrations than nonobese participants, irrespective of metabolic health status. Finally, we postulated that, for a given BMI category, HIV-infected metabolically unhealthy men would have higher circulating levels of markers of systemic inflammation and insulin resistance and a greater imbalance of adipokines than HIV-uninfected metabolically unhealthy men, suggesting that HIV infection and/or ART amplifies the adverse immuno-metabolic consequences of traditional metabolic dysregulation.

## 2. Methods

### 2.1. Study Population

The MACS is an ongoing, multicenter (Pittsburgh, Pennsylvania; Baltimore, Maryland/Washington, DC; Chicago, Illinois; and Los Angeles, California, USA), prospective, observational cohort study of men who have sex with men, in which participants return semiannually for a standardized interview, clinical evaluations, laboratory tests, and specimen storage. We conducted a cross-sectional analysis of 470 men living with HIV and 368 HIV-uninfected men enrolled in the MACS Cardiovascular 2 (CVD2) substudy, for which both biomarker levels and metabolic health data were available [18]. We restricted the analysis among HIV-infected men to those receiving ART and with plasma HIV-1 RNA < 50 copies/mL (Ultrasensitive Amplicor HIV-1 Monitor Assay, Roche Diagnostics, Pleasanton, California, USA) at the visit of interest. Inclusion criteria for the CVD2 substudy included age 40–70 years, weight less than 300 pounds, and no history of prior cardiac surgery, percutaneous transluminal coronary angioplasty, or stent placement. All participants provided written informed consent; all protocols were approved by local Institutional Review Boards of participating sites, and the study was conducted in accordance with the Declaration of Helsinki.

### 2.2. Metabolic Definitions

We defined metabolic health using the National Cholesterol Education Program Adult Treatment Panel III definition, which requires the presence of two or fewer of the following: systolic blood pressure ≥ 130 mmHg, diastolic blood pressure ≥ 85 mmHg, triglycerides ≥ 150 mg/dL, high-density lipoprotein cholesterol < 40 mg/dL, fasting plasma glucose ≥ 100 mg/dL, and waist circumference > 102 cm [16]. This definition was selected due to its frequent use and ease of clinical implementation. Use of blood pressure-, lipid-, or glucose-lowering medications were considered evidence of the corresponding diagnosis. BMI categories were defined as <25.0, 25.0–29.9, 30.0–34.9, and ≥35.0 kg/m^2^. In previous analyses, we created four categories combining metabolic status and BMI categories: MHO (BMI > 30 kg/m^2^ with metabolic health), metabolically unhealthy obese (BMI > 30 kg/m^2^ and >2 of the metabolic criteria), metabolically healthy nonobese (BMI < 30 kg/m^2^ with metabolic health), and metabolically unhealthy nonobese (BMI < 30 kg/m^2^ and >2 of the metabolic criteria) [17]. For our analysis, metabolic health status, assessed by HIV serostatus, BMI category, and type of metabolic disturbance, was acquired from the MACS visit closest to the visit with CVD2 biomarker data (January 2010 to December 2012).

### 2.3. Other Measurements

Age, race, level of education, smoking history, medication use, and clinical diagnosis history were assessed by self-report, with current metabolic diagnoses confirmed by laboratory or medication use. AIDS-defining illness and other clinical events were confirmed via medical record review. Chronic hepatitis C virus (HCV) infection was defined as plasma HCV RNA positivity. Height and weight were measured using standardized procedures and used to calculate the BMI in kg/m^2^. Waist circumference (cm) at the iliac crest was measured using a standardized protocol [19]. All sociodemographic and medical data were obtained at the visit that was closest (<1 year) in time to the visit with both CVD biomarkers and complete metabolic data from October 1, 2009 to March 31, 2014. CD4^+^ T lymphocyte subsets were measured using standardized flow cytometry, and CD4^+^ T lymphocyte/mL count nadir was defined as the lowest count prior to and including the most recent visit with complete metabolic data and CVD biomarkers.

### 2.4. Laboratory Analysis

Levels of circulating markers of systemic inflammation, such as IL-6, high-sensitivity C-reactive protein (hs-CRP), soluble TNF receptors (sTNFR) I and II, homeostatic model assessment of insulin resistance (HOMA-IR), soluble CD14 (sCD14), and soluble CD163 (sCD163), and AT function markers, including adiponectin and leptin, were compared by metabolic phenotype and BMI category.

Levels of IL-6 (serum) were measured using chemiluminescent ELISA (R&D Systems, Minneapolis, MN) with a lower limit of detection (LLOD) of 0.5 pg/mL and an interassay coefficient of variation (CV) range of 6.6–12.5%. Levels of hs-CRP (serum) were measured by nephelometry (LLOD 0.2 *μ*g/mL; interassay CV range 3.0%–6.2%). Levels of sTNFRI and sTNFRII (EDTA plasma) were measured via multiplexing using a Milliplex soluble cytokine receptor panel (Millipore, Billerica, MA). The interassay CVs were 4.6% to 10.8% for sTNFRI and 4.2% to 7.9% for sTNFRII. Levels of sCD163 (serum) and sCD14 (plasma) were measured by enzyme-linked immunosorbent assay (ELISA; R&D Systems; sCD163 interassay CV range 1.71%–5.80%; sCD14 interassay CV range 3.96%–5.22%). All blood samples were processed in batch at the University of Vermont Laboratory for Clinical Biochemistry Research Lab (Burlington, VT) under the direction of Dr. Russell Tracy.

Fasting insulin and glucose concentrations were measured from serum, collected under standardized protocols and stored at −70°C by the Heinz Laboratory (Pittsburgh, Pennsylvania, USA). Insulin was measured using a radioimmunoassay technique (Linco Research, St. Charles, Missouri, USA) with a LLOD of 0.2 *μ*U/mL and a CV of 2.6%. Fasting glucose concentrations were measured using a combined hexokinase/glucose-6-phosphate dehydrogenase method (CV 1.8%). Fasting glucose and insulin concentrations were used to calculate HOMA-IR values according to the formula HOMA − IR = (fasting glucose(mmol/L) × fasting insulin(*μ*U/mL))/22.5.

Adiponectin and leptin were measured at the University of Vermont Laboratory for Clinical Biochemistry Research Lab (Burlington, VT) by ELISA (R&D Systems, Minneapolis, MN) on plasma samples that had been stored at −70°C. The LLOD for adiponectin assay was 390 ng/mL, with an interassay CV of 5.3–10.8%; for leptin, the corresponding values were 1300 pg/mL and 5.9–6.8%.

### 2.5. Imaging Protocols

Noncontrast CT was performed using multidetector scanners at each site. Visceral adipose tissue (VAT) area (cm^2^) was quantified from a single CT slice obtained at the level of the umbilicus, as previously described [20]. The amount of abdominal subcutaneous adipose tissue (SAT) was calculated by subtracting VAT fat from total abdominal fat. CT scans were read centrally by a blinded reader (Los Angeles Biomedical Research Institute [LA Biomed], Torrance, CA).

### 2.6. Statistical Analysis

A descriptive analysis of demographic and clinical characteristics was generated and stratified by HIV serostatus. Continuous variables are expressed as median with interquartile range. Wilcoxon rank-sum tests and Pearson *χ* ^2^ tests compared continuous and categorical characteristics, respectively. All statistical tests are two-sided (*α* = 0.05).

Biomarker concentrations were compared by metabolic health status using Wilcoxon rank-sum tests, stratified by BMI category and by HIV serostatus. Among HIV-infected men population stratified by BMI categories, Poisson regression with robust variance determined associations between metabolic health and circulating inflammatory biomarker concentrations after adjusting for factors known to be associated with metabolic health from our previous analysis (age, race, smoking, HCV infection, protease inhibitor [PI] use, thymidine analog nucleoside reverse transcriptase inhibitor [NRTI] use, and current CD4^+^ T lymphocyte count) [17]. Multiple imputation was used to complete missing covariate data (2%). Five imputation data sets were created using multivariable normal model, including all variables in the Poisson model. The final estimates of the association between metabolic health and factors examined were obtained by averaging the estimates from the five imputation data sets.

## 3. Results

### 3.1. Participant Population

Of the 368 HIV-uninfected and 470 HIV-infected participants receiving ART with HIV-1 RNA less than 50 copies/mL, HIV-infected men were younger and more likely to be of nonwhite race, have a high school or lower education, have a slightly lower median BMI and waist circumference, and to have dyslipidemia with elevated triglycerides and low HDL cholesterol, metabolic syndrome, and HCV coinfection. Rates of diabetes mellitus and hypertension were similar by HIV serostatus (Table 1).

Among HIV-infected men, median time since HIV diagnosis was 16.4 years, with median current CD4^+^ T lymphocyte count 609 cells/mL (Table 1). In addition to a high prevalence of virologic suppression at the visit associated with metabolic health determination, participants overall exhibited durable virologic suppression, with HIV-1 RNA less than 50 copies/mL at nearly 90% of visits in the 5 years preceding the visit of metabolic health determination. In total, 14.7% of participants had a history of a diagnosis of AIDS. Current ART regimens included PI 48% (atazanavir 41%, darunavir 30%, and lopinavir 17%), non-NRTI 52% (efavirenz 67%), and integrase strand transfer inhibitor (INSTI) 19%, with 95% of men receiving regimens containing one or more NRTIs.

### 3.2. Differences in Clinical and Laboratory Parameters by HIV Serostatus and Metabolic Health Status among Normal Weight Men

Among HIV-uninfected normal weight (BMI < 25 kg/m^2^) men, metabolically healthy men had a significantly higher median adiponectin concentration (*P* = 0.001) and significantly lower median VAT area (*P* = 0.002) and HOMA-IR values (*P* = 0.03) than HIV-uninfected normal weight metabolically unhealthy men (Table 2). Age, thigh SAT, HOMA-IR, and concentrations of IL-6, hs-CRP, sTNFR I and II, sCD163, and sCD14 did not differ significantly by metabolic health status among HIV-uninfected normal weight men.

Similarly, HIV-infected normal weight metabolically healthy men had significantly higher median adiponectin concentrations (*P* < 0.001), less VAT area (*P* < 0.001), greater SAT area (*P* = 0.001), and lower HOMA-IR values (*P* < 0.001) and circulating concentrations of IL-6 (*P* = 0.03), sTNFRI (*P* < 0.001), and sTNFRII (*P* = 0.001) than HIV-infected normal weight metabolically unhealthy men. Concentrations of hs-CRP, sCD163, sCD14, and leptin did not differ significantly. Overweight men with BMI 25.0–29.9 kg/m^2^ showed similar results to normal weight men within their respective HIV serostatus (data not shown).

### 3.3. Differences in Clinical and Laboratory Parameters by HIV Serostatus and Metabolic Health Status among Obese Men

HIV-uninfected men with MHO had significantly lower circulating IL-6 concentrations (*P* = 0.02), HOMA-IR values (*P* < 0.001), and VAT area (*P* = 0.004) than HIV-uninfected obese metabolically unhealthy men (Table 3). Men with MHO also had significantly higher adiponectin concentrations than obese metabolically unhealthy men (*P* = 0.02). SAT area and circulating concentrations of hs-CRP, sTNFRI, sTNFRII, sCD163, sCD14, and leptin did not differ significantly by metabolic health status among HIV-uninfected obese men.

HIV-infected men with MHO had significantly higher adiponectin concentrations (*P* = 0.009) and significantly lower VAT area (*P* = 0.001) and HOMA-IR values (*P* < 0.001) than metabolically unhealthy obese men, mirroring that seen among HIV-uninfected obese men. SAT area and circulating IL-6, hs-CRP, sTNFRI, sTNFRII, CD163, sCD14, and leptin concentrations did not differ by metabolic health status among HIV-infected obese men.

### 3.4. Comparison of Inflammatory Biomarkers by Metabolic Health Status and BMI Category by HIV Serostatus

Among normal weight metabolically healthy men, the HIV-infected men had higher concentrations of IL-6 (*P* = 0.03), hs-CRP (*P* = 0.001), sTNFRII (*P* = 0.001), CD163 (*P* = 0.002), CD14 (*P* < 0.001), and HOMA-IR (*P* < 0.001) and lower concentrations of adiponectin (*P* = 0.02) and greater VAT area (*P* = 0.01) and less SAT area (*P* < 0.001) than their HIV-uninfected counterparts (Table 4).

Among normal weight metabolically unhealthy men, the HIV-infected men had higher concentrations of sTNFRI (*P* = 0.003), sTNFRII (*P* = 0.001), CD163 (*P* = 0.013), CD14 (*P* < 0.001), greater VAT area (*P* = 0.024), and less SAT area (*P* < 0.001) than their HIV-uninfected counterparts. Among obese men regardless of metabolic health status, HIV-infected men had higher sCD14 and less SAT area (all *P* ≤ 0.01).

### 3.5. Associations between Inflammatory Biomarker Concentrations and Metabolic Health Status among HIV-Infected Men

We further investigated the unique relationship between having less systemic inflammation and metabolic health among HIV-infected normal weight men in multivariable Poisson regression with robust variance stratified by BMI categories. Factors associated with metabolic health in our previous analysis were adjusted, including age, race, smoking, HCV infection, PI use, thymidine analog NRTI use, and current CD4^+^ T lymphocyte count [17]. Among HIV-infected men with normal weight, lower log hs-CRP, sTNFRI, sTNFRII, and HOMA-IR values and higher adiponectin concentrations were associated with metabolic health (Table 5). Among HIV-infected obese men, only higher adiponectin concentrations and lower HOMA-IR values were associated with metabolic health. No association was observed for IL-6, sCD14, sCD163, or leptin concentrations. Additional adjustment for visceral adiposity did not significantly alter results (data not shown).

## 4. Discussion

Among men living with HIV receiving suppressive ART, those who were normal weight and metabolically healthy demonstrated less systemic inflammation than metabolically unhealthy men. This difference in inflammation by metabolic health status was not apparent among normal weight HIV-uninfected men nor among obese men, regardless of HIV serostatus. To our knowledge, our study is the first to assess associations between systemic inflammatory perturbations and metabolic health by HIV serostatus and BMI.

Persons living with HIV demonstrate metabolic disturbances beyond the presence of traditional CVD risk factors that may be linked to chronic inflammatory processes [21]. Increased concentrations of hs-CRP, IL-6, and d-dimer were also independently associated with CVD risk in the SMART Study, and measurement of these markers could improve prediction of CVD risk among persons living with HIV [22]. The AIDS Clinical Trial Group (ACTG) substudy A5224s demonstrated that higher pre-ART hs-CRP and time-updated TNF-*α*, sTNFRI, and sTNFRII concentrations were significantly associated with increased risk of non-AIDS-defining events (including myocardial infarction and pulmonary embolism), non-AIDS-defining malignancies, and diabetes mellitus, independently of CD4^+^ T lymphocyte count over 96 weeks of follow-up [23].

Among our participants, HIV-infected normal weight metabolically unhealthy men had significantly higher concentrations of IL-6, sTNFRI, and sTNFRII compared to HIV-infected normal weight metabolically healthy men. These differences in biomarker concentrations by metabolic health status did not exist among normal weight HIV-uninfected men nor among obese men regardless of HIV serostatus. Furthermore, the differences in biomarker levels among normal weight men by HIV serostatus, which were not observed among obese men of either HIV serostatus (Table 4), emphasize that the magnitude and sequelae of increased systemic inflammation in HIV may be more evident among normal weight and overweight men than among obese men, in whom obesity-related inflammation may outweigh and/or obfuscate HIV-related increases in inflammation.

Adiponectin and leptin are adipose tissue-derived peptides that are associated with myocardial infarction in the general population [24, 25]. Adiponectin increases AMP-kinase and peroxisome proliferator activated receptor- (PPAR-) *α* activities, fatty acid oxidation, and glucose uptake, leading to increased insulin sensitivity [26]. Leptin is a protein involved in weight regulation and energy homeostasis, and leptin levels are altered in obese individuals as well as in those with lipodystrophy [27]. Importantly, adipokine imbalance might contribute to subclinical coronary atherosclerosis and glucose disorders in people with HIV: leptin concentrations correlate positively with both insulin concentrations and HOMA-IR in lipodystrophic patients with HIV, and adiponectin concentrations correlate negatively with these parameters [28]. In a MACS cohort substudy that included 493 HIV-infected and 250 HIV-uninfected men, adiponectin concentrations were lower among men with HIV after adjusting for traditional CVD risk factors (*P* < 0.0001), and lower adiponectin concentrations were associated with increased odds of coronary stenosis (*P* < 0.007) [15]. Our findings are consonant with these prior findings, in that adiponectin concentrations were significantly higher among metabolically healthy vs metabolically unhealthy men, regardless of BMI category or HIV serostatus, whereas leptin concentrations did not vary by metabolic health category or HIV serostatus within a given BMI category, similar to previous findings [15]. However, metabolically healthy normal weight HIV-infected men in our cohort had lower adiponectin, higher VAT area (associated with insulin resistance) [29], and higher HOMA-IR values than HIV-uninfected men in the same metabolic category, supporting the notion of greater adipose tissue dysfunction in these men. Of note, oxidative stress plays a major role in inflammation and may contribute to the development of CVD [30]. Measurement of oxidized lipoproteins and total lipoprotein particle number and size may help clarify the mechanisms of inflammation and the origin of coronary plaque in men living with HIV and will be explored in future studies.

Of note, the potential mechanisms driving the observed differences between HIV-infected and HIV-uninfected MACS men cannot be gleaned from this cross-sectional analysis. However, given the high rates of exposure to older ART agents, including thymidine analogue NRTIs (tNRTI, which have been associated with inflammation, mitochondrial toxicity, adipose tissue dysfunction, and hepatic steatosis), in this group of HIV-infected men, we hypothesize these differences may be derived, in part, by tNRTI-associated tissue dysfunction and immuno-metabolic disturbances. Further studies including tissue samples are needed to fully address this question but are beyond the scope of this project.

Related to tNRTI exposure, abdominal subcutaneous adipose tissue area was significantly lower in HIV-infected MACS men compared to HIV-uninfected men, regardless of BMI category. We did not additionally include a clinical assessment of lipodystrophy status, as the metabolic health definition and CT body composition determinations provide more consistent and objective platforms for assessment than a clinician's subjective lipodystrophy assessment.

This study has several limitations. First, our assessment of metabolic health status was cross-sectional. However, as noted in the methods, when comparing the most recent visit to metabolic health status over the last 5 years, results were comparable and using the most current visit allowed for presentation of data per person rather than in person-years. Second, the types of metabolic disturbances used to determine metabolic health status differed in prevalence by HIV serostatus, with HIV-infected men more likely to meet criteria for lipid abnormalities. Acknowledging that each Adult Treatment Panel III criteria may not contribute equally to cardiometabolic outcomes, our next planned step is to conduct a longitudinal study to determine how the currently observed differences in metabolic health and immuno-metabolic biomarker profiles affect clinical outcomes by HIV serostatus and by BMI category. Thirdly, creation of numerous categories left subject numbers relatively small in certain instances. These results should be addressed with a larger population. Fourth, this analysis may not be generalizable to women living with HIV or populations outside of the United States, although analyses in these populations are planned. Finally, the Adult Treatment Panel III criteria for MHO, while common in the literature and applicable to the majority of MACS men, do not incorporate waist circumference measurements that are race- or ethnicity-specific. Despite these limitations, strengths of this analysis include our large sample size, detailed participant characterization, HIV-uninfected control group with similar risk factor profiles, and diverse geographic makeup.

## 5. Conclusions

HIV-infected normal weight metabolically healthy men have less systemic inflammation, immune activation, and adipokine disruption than metabolically unhealthy normal weight HIV-infected men. These differences in inflammatory and immuno-metabolic parameters by metabolic health status were not apparent among normal weight HIV-uninfected men nor among obese men regardless of HIV serostatus. These findings, including the fact that levels of inflammation were higher among metabolically healthy HIV-infected men compared to otherwise similar HIV-uninfected men, highlight the disproportionate contribution of HIV-associated inflammation and immune activation to metabolic disease among normal weight HIV-infected men. Future studies to determine the mechanisms by which inflammation and immune dysregulation contribute to or are affected by specific metabolic disturbances are needed; such inquiries have the potential to lead to the development of targeted interventions that can reduce inflammation levels and thereby mitigate the effects of metabolic risk factors.

## Figures and Tables

**Table 1:** Demographic and clinical characteristics.

Percent or median (IQR)	HIV (+) (*n* = 470)	HIV (−) (*n* = 368)	*P* value
Age (years)	53 (48–59)	55 (50–62)	<0.001
Nonwhite race (%)	41.5	32.6	0.01
High school education (%)	21.5	14.9	0.02
Current smoking (%)	25.7	21.6	0.17
BMI (kg/m^2^)	25.7 (23.3–28.3)	26.5 (24–29.9)	0.001
Waist circumference	94.5 (88–102)	97 (90–107)	0.001
Hypertension (%)	48.6	44.1	0.21
Dyslipidemia (%)	85.9	76.2	<0.001
Diabetes mellitus (%)	13.4	9.7	0.11
Metabolic syndrome (%)	35.5	26.9	0.01
HCV coinfection (%)	8.4	4.1	0.01
Time since HIV diagnosis (years)	16.4 (8.5–25.7)	—	—
Most recent CD4^+^ T cell count (cells/*μ*L)	609 (449–793)	—	—
Nadir CD4^+^ T cell count (cells/*μ*L)	239 (134–324)	—	—
AIDS diagnosis (%)	14.7	—	—

HCV: hepatitis C virus; IQR: interquartile range.

**Table 2:** Differences in clinical and laboratory parameters by metabolic health status: BMI < 25 kg/m^2^.

	Metabolically healthy		Metabolically unhealthy		*P* value
	*n*	Median (IQR)	*n*	Median (IQR)	
**HIV−**					
Age (years)	124	55 (50, 62)	9	59 (54, 62)	0.41
IL-6 (pg/mL)	115	1.1 (0.6, 1.8)	9	1.1 (1.0, 1.6)	0.55
hs-CRP (*μ*g/mL)	118	0.8 (0.4, 1.3)	9	1.0 (0.3, 1.2)	0.95
sTNFRI (pg/mL)	118	1075 (914, 1317)	9	848 (699, 1319)	0.25
sTNFRII (pg/mL)	118	5496 (4737, 6595)	9	5904 (3912, 6065)	0.34
sCD163 (ng/mL)	113	511 (418, 674)	9	549 (323, 655)	0.51
sCD14 (ng/mL)	111	1341 (1126, 1549)	9	122 (1158, 1425)	0.42
Adiponectin (ng/mL)	117	9441 (6955, 13399)	9	3654 (3111, 5020)	0.001
Leptin (pg/mL)	117	2958 (1556, 4924)	9	3436 (3082, 3972)	0.24
Abdominal VAT area (cm^2^)	123	87 (54, 118)	9	145 (114, 165)	0.002
Thigh subcutaneous fat area (cm^2^)	124	35 (26, 44)	9	32 (26, 35)	0.42
HOMA-IR (mmol/L × *μ*U/mL)	107	2.0 (2.0, 3.0)	9	3.0 (2.0, 4.0)	0.03

**HIV+**					
Age (years)	164	53 (49, 59)	42	57 (52, 62)	0.02
IL-6 (pg/mL)	159	1.3 (0.8, 2.1)	40	1.9 (1.1, 2.3)	0.03
hs-CRP (*μ*g/mL)	159	1.1 (0.5, 2.6)	40	1.6 (0.8, 2.9)	0.06
sTNFRI (pg/mL)	160	1151 (948, 1421)	41	1416 (1171, 1811)	<0.001
sTNFRII (pg/mL)	160	6302 (5307, 7531)	41	7126 (6343, 10,467)	0.001
sCD163 (ng/mL)	150	601 (451, 807)	37	699 (577, 893)	0.12
sCD14 (ng/mL)	151	1728 (1517, 2015)	35	1807 (1539, 2204)	0.18
Adiponectin (ng/mL)	158	8375 (4850, 12,397)	40	4392 (2375, 8370)	<0.001
Leptin (pg/mL)	158	2733 (1540, 5208)	40	3995 (1764, 7720)	0.07
Abdominal VAT area (cm^2^)	164	108 (62, 154)	41	182 (139, 217)	<0.001
Thigh subcutaneous fat area (cm^2^)	164	18 (7, 31)	41	10 (4, 18)	0.001
HOMA-IR (mmol/L × *μ*U/mL)	135	3.0 (2.0, 3.0)	40	4.0 (3.0, 6.0)	<0.001

HOMA-IR: homeostatic model assessment of insulin resistance; hs-CRP: high-sensitivity C-reactive protein; IL-6: interleukin-6; IQR: interquartile range; sCD163: soluble CD163; sCD14: soluble CD14; sTNFRI-II: soluble tumor necrosis factor receptor I-II; VAT: visceral adipose tissue.

**Table 3:** Differences in clinical and laboratory parameters by metabolic health status: BMI > 30 kg/m^2^.

	Metabolically healthy		Metabolically unhealthy		*P* value
	*n*	Median (IQR)	*n*	Median (IQR)	
**HIV−**					
Age (years)	31	53 (45, 62)	60	56 (51, 62)	0.1
IL-6 (pg/mL)	30	1.4 (1.0, 1.8)	53	2.0 (1.5, 3.0)	0.02
hs-CRP (*μ*g/mL)	30	1.4 (0.9, 3.8)	55	2.7 (0.9, 5.2)	0.39
sTNFRI (pg/mL)	30	1240 (1017, 1309)	55	1275 (1056, 1485)	0.45
sTNFRII (pg/mL)	30	6120 (4976, 6961)	55	6479 (5511, 7818)	0.27
sCD163 (ng/mL)	30	597 (424, 705)	52	628 (503, 813)	0.11
sCD14 (ng/mL)	30	1250 (1078, 1433)	53	1274 (1133, 1447)	0.49
Adiponectin (ng/mL)	30	6464 (4916, 7964)	54	5025 (3862, 6657)	0.02
Leptin (pg/mL)	30	13887 (10703, 21452)	54	18829 (12350, 28725)	0.05
Abdominal VAT area (cm^2^)	31	190 (140, 263)	60	262 (202, 325)	0.004
Thigh subcutaneous fat area (cm^2^)	31	10.5 (7.6, 17.4)	60	11.5 (9.4, 14.6)	0.82
HOMA-IR (mmol/L × *μ*U/mL)	29	3.0 (2.0, 4.0)	53	5.0 (4.0, 8.0)	<0.001

**HIV+**					
Age (years)	23	53 (47, 59)	62	52 (47, 57)	0.53
IL-6 (pg/mL)	23	1.6 (1.0, 2.3)	58	1.8 (1.3, 2.5)	0.27
hs-CRP (*μ*g/mL)	22	1.4 (0.8, 2.8)	59	1.7 (0.9, 3.0)	0.36
sTNFRI (pg/mL)	23	1226 (1023, 1499)	59	1150 (946, 1499)	0.50
sTNFRII (pg/mL)	23	5751 (5008, 7785)	59	6271 (5334, 7684)	0.36
sCD163 (ng/mL)	23	646 (513, 793)	58	686 (486, 835)	0.76
sCD14 (ng/mL)	23	1453 (1336, 1672)	58	1495 (1240, 1771)	0.73
Adiponectin (ng/mL)	23	6167 (3876, 8089)	60	4359 (3044, 5910)	0.009
Leptin (pg/mL)	23	14262 (8409, 16957)	60	13318 (10965, 20808)	0.64
Abdominal VAT area (cm^2^)	23	183 (134, 242)	62	267 (205, 352)	0.001
Thigh subcutaneous fat area (cm^2^)	23	62 (41, 94)	62	44 (26, 74)	0.09
HOMA-IR (mmol/L × *μ*U/mL)	22	3.0 (2.0, 4.0)	58	6.0 (4.0, 10.0)	<0.001

HOMA-IR: homeostatic model assessment of insulin resistance; hs-CRP: high-sensitivity C-reactive protein; IL-6: interleukin-6; IQR: interquartile range; sCD163: soluble CD163; sCD14: soluble CD14; sTNFRI-II: soluble tumor necrosis factor receptor I-II; VAT: visceral adipose tissue.

**Table 4:** Biomarkers concentrations by metabolic health status and HIV serostatus.

Biomarkers	Metabolically healthy					Metabolically unhealthy				
	HIV (−) (*n*)	HIV (−)^∗^	HIV (+) (*n*)	HIV (+)^∗^	*P* value	HIV (−) (*n*)	HIV (−)^∗^	HIV (+) (*n*)	HIV (+)^∗^	*P* value
BMI < 25 kg/m^2^										
IL-6 (pg/mL)	115	1.1 (0.6–1.8)	159	1.3 (0.8–2.1)	0.034	9	1.1 (1–1.6)	40	1.9 (1.1–2.3)	0.121
hs-CRP (*μ*g/mL)	118	0.8 (0.4–1.3)	159	1.1 (0.5–2.6)	0.001	9	1 (0.3–1.2)	40	1.6 (0.8–2.9)	0.054
sTNFRI (pg/mL)	118	1075 (914–1317)	160	1151 (948–1421)	0.146	9	848 (699–1319)	41	1416 (1171–1811)	0.003
sTNFRII (pg/mL)	118	5496 (4737–6595)	160	6302 (5307–7531)	0.001	9	5904 (3912–6065)	41	7126 (6343–10467)	0.001
sCD 163 (ng/mL)	113	511 (418–674)	150	601 (451–807)	0.002	9	549 (323–655)	37	699 (577–893)	0.013
sCD 14 (ng/mL)	111	1341 (1126–1549)	151	1728 (1517–2015)	<0.001	9	1221 (1158–1425)	35	1807 (1539–2204)	<0.001
Adiponectin (ng/mL)	117	9441 (6955–13399)	158	8375 (4850–12397)	0.025	9	3654 (3111–5020)	40	4392 (2375–8370)	0.928
Leptin (pg/mL)	117	2958 (1556–4924)	158	2733 (1540–5208)	0.977	9	3436 (3082–3972)	40	3995 (1764–7720)	0.969
Abdominal VAT area (cm^2^)	123	87 (54–118)	164	108 (62–154)	0.006	9	145 (114–165)	41	182 (139–217)	0.024
Thigh subcutaneous fat area (cm^2^)	124	35 (26–44)	164	18 (7–31)	<0.001	9	32 (26–35)	41	10 (4–18)	<0.001
HOMA-IR (mmol/L × *μ*U/mL)	107	2 (2-3)	135	3 (2-3)	<0.001	9	3 (2–4)	40	4 (3–6)	0.056

BMI > 30 kg/m^2^										
IL-6 (pg/mL)	30	1.4 (1–1.8)	23	1.6 (1–2.3)	0.747	53	2 (1.5–3)	58	1.8 (1.3–2.5)	0.277
hs-CRP (*μ*g/mL)	30	1.4 (0.9–3.8)	22	1.4 (0.8–2.8)	0.42	55	2.7 (0.9–5.2)	59	1.7 (0.9–3)	0.179
sTNFRI (pg/mL)	30	1240 (1017–1309)	23	1226 (1023–1499)	0.74	55	1275 (1056–1485)	59	1150 (946–1499)	0.269
sTNFRII (pg/mL)	30	6120 (4976–6961)	23	5751 (5008–7785)	0.964	55	6479 (5511–7818)	59	53	0.816
sCD 163 (ng/mL)	30	597 (424–705)	23	646 (513–793)	0.116	52	628 (503–813)	58	686 (486–835)	0.484
sCD 14 (ng/mL)	30	1250 (1078–1433)	23	1453 (1336–1672)	0.01	53	1274 (1133–1447)	58	1495 (1240–1771)	0.002
Adiponectin (ng/mL)	30	6464 (4916–7964)	23	6167 (3876–8089)	0.74	54	5025 (3862–6657)	60	4359 (3044–5910)	0.135
Leptin (pg/mL)	30	13887 (10703–21452)	23	14262 (8409–16957)	0.66	54	18829 (12350–28725)	60	13318 (10965–20808)	0.028
Abdomen visceral fat area (cm^2^)	31	190 (140–263)	23	183 (134–242)	0.766	60	262 (202–325)	62	267 (205–352)	0.456
Thigh subcutaneous fat area (cm^2^)	31	83 (69–123)	23	62 (41–94)	0.004	60	82 (61–101)	62	44 (26–74)	<0.001
HOMA-IR (mmol/L × *μ*U/mL)	29	3 (2–4)	22	3 (2–4)	0.718	53	5 (4–8)	58	6 (4–10)	0.422

^∗^Median (interquartile range); HOMA-IR: homeostatic model assessment of insulin resistance; hs-CRP: high-sensitivity C-reactive protein; IL-6: interleukin-6; IQR: interquartile range; sCD163: soluble CD163; sCD14: soluble CD14; sTNFRI-II: soluble tumor necrosis factor receptor I-II; VAT: visceral adipose tissue.

**Table 5:** Adjusted prevalence ratio for metabolic health among HIV+ men stratified by BMI categories per log biomarker increase.

Biomarkers	BMI < 25 PR (95% CI)	*P* value	BMI > 30 PR (95% CI)	*P* value
IL-6	0.92 (0.84, 1.01)	0.07	0.67 (0.36, 1.27)	0.222
hs-CRP	0.92 (0.87, 0.98)	0.01	0.78 (0.52, 1.16)	0.222
sTNFRI	0.73 (0.61, 0.87)	<0.001	1.25 (0.46, 3.42)	0.662
sTNFRII	0.68 (0.54, 0.85)	0.001	0.48 (0.13, 1.81)	0.28
sCD163	0.92 (0.77, 1.1)	0.35	1.05 (0.42, 2.62)	0.916
sCD14	0.89 (0.79, 1.01)	0.07	1.47 (1, 2.17)	0.052
Adiponectin	1.27 (1.12, 1.43)	<0.001	2.36 (1.35, 4.13)	0.003
Leptin	0.93 (0.84, 1.03)	0.15	0.87 (0.58, 1.3)	0.498
HOMA-IR	0.69 (0.55, 0.86)	<0.001	0.22 (0.08, 0.59)	0.003

BMI: body mass index; CI: confidence interval; IL-6: interleukin-6; hs-CRP: high-sensitivity C-reactive protein; sTNFRI-II: soluble tumor necrosis factor receptor I-II; sCD163: soluble CD163; sCD14: soluble CD14; HOMA-IR: homeostatic model assessment of insulin resistance.

## Data Availability

The data used to support the findings of this study are included within the article.

## Supplementary Materials

Supplementary Materials The supplementary tables provide data about Biomarker Concentrations by ART regimens in response to Review Report #1 and Review Report #3.
