# Supplementary material for: Systemic Inflammation Characterizes Lack of Metabolic Health in Nonobese HIV-Infected Men
Source: Mediators Inflamm. 2018 Sep 25;2018:5327361. doi: 10.1155/2018/5327361 (PMC6176328; doi:10.1155/2018/5327361)
Supplement: Supplementary Materials — The supplementary tables provide data about Biomarker Concentrations by ART regimens in response to Review Report #1 and Review Report #3. [file 5327361.f1.pdf]

Supplementary Table 1. Biomarkers concentrations by Antiretroviral Regimen

Biomarkers	PI(n)	PI*	NNRTI(n)	NNRTI*	INSTI(n)	INSTI*
Age (years)	227	53(48-59)	245	53(49-59)	87	55(50-59)
IL-6 (pg/ml)	221	1.5(1.1-2.4)	238	1.4(0.9-2.2)	82	1.8(1.1-2.9)
CRP (ug/ml)	222	1.1(0.7-2.5)	238	1.3(0.7-2.7)	82	1.3(0.7-2.8)
sTNFRI (pg/ml)	223	1162(976-1499)	236	1222(982-1519)	85	1208(1022-1516)
sTNFRII (pg/ml)	223	6588(5425-8131)	236	6220(5283-7578)	85	7079(5802-8717)
sCD 163 (ng/ml)	217	693(535-855)	226	623(476-796)	79	734(533-978)
sCD 14 (ng/ml)	214	1587(1360-1859)	225	1707(1502-1993)	77	1583(1375-1925)
Adioponectin (ng/ml)	223	5755(3553-9220)	237	6391(4082-9949)	82	5845(3369-9913)
Leptin (pg/ml)	223	6118(2980-11660)	237	4847(2260-9173)	82	6034(2554-14818)
Abdominal VAT area (cm2)	226	159(99-218)	245	160(103-220)	87	178(104-228)
Thigh subcutaneous fat area (cm2)	226	24(10-46)	245	23(10-38)	87	20(8-51)
HOMA-IR (mmol/L x uU/ml)	196	4(2-6)	210	3(2-4)	72	3(2-5)

*Median (interquartile range) or percent; HOMA-IR: homeostatic model assessment of insulin resistance; hs-CRP: high-sensitivity C-reactive protein; IL-6: Interleukin-6; INSTI=integrase strand transfer inhibitors IQR: interquartile range; sCD163: soluble CD163; sCD14: soluble CD14; NNRTI=Non-Nucleoside Reverse Transcriptase Inhibitor; PI=Protease inhibitor; sTNFRI-II: Soluble tumor necrosis factor receptor I-II; VAT: Visceral adipose tissue

Supplementary Table 2. Biomarkers concentrations by Most Common Antiretroviral Drugs

CVD2 markers	*Lopinavir(n)*	*Lopinavir**	*Atazanavir(n)*	*Atazanavir**	*Darunavir(n)*	*Darunavir**	*Efavirenz(n)*	*Efavirenz**
Age at CVD2 CT-scan(years)	38	53(48-57)	93	52(47-58)	67	55(48-59)	164	52(48-58)
IL-6 (pg/ml)	38	1.5(1-2)	89	1.4(1-2.4)	65	1.6(1.1-2.6)	160	1.3(0.9-2.2)
CRP (ug/ml)	38	1.1(0.8-2.2)	90	1.1(0.6-2.5)	65	1.4(0.7-2.6)	160	1.4(0.7-2.9)
sTNFRI (pg/ml)	38	1116(888-1411)	90	1122(946-1486)	66	1244(1023-1522)	158	1197(973-1462)
sTNFRII (pg/ml)	38	6222(4892-7443)	90	6330(5461-7873)	66	6884(5775-8717)	158	6093(5262-7429)
sCD 163 (ng/ml)	38	643(533-835)	89	661(520-798)	62	745(616-970)	152	574(444-745)
sCD 14 (ng/ml)	38	1774(1493-2015)	90	1568(1326-1819)	59	1582(1353-1952)	152	1723(1518-1982)
Adioponectin (ng/ml)	38	6287(4493-9156)	90	5291(3548-9110)	66	5949(3455-9646)	160	6469(4245-10144)
Leptin (pg/ml)	38	6354(3136-11448)	90	6717(3523-12883)	66	6804(3052-11953)	160	4853(2107-8705)
Abdominal VAT area (cm2)	38	143(88-235)	93	154(106-226)	67	178(134-217)	164	150(90-204)
Thigh subcutaneous fat area (mm2)	38	31(6-46)	93	26(13-49)	67	21(9-45)	164	25(13-40)
HOMA-IR (mmol/L x uU/ml)	35	3(3-5)	81	4(3-6)	55	3(2-5)	140	3(2-4)

*Median (interquartile range) or percent; HOMA-IR: homeostatic model assessment of insulin resistance; hs-CRP: high-sensitivity C-reactive protein; IL-6: Interleukin-6; sCD163: soluble CD163; sCD14: soluble CD14; sTNFRI-II: Soluble tumor necrosis factor receptor I-II; VAT: Visceral adipose tissue
